# Placental Tissues as Biomaterials in Regenerative Medicine

**DOI:** 10.1155/2022/6751456

**Published:** 2022-04-21

**Authors:** Annelise Roy, Morgan Mantay, Courtney Brannan, Sarah Griffiths

**Affiliations:** Research and Development, StimLabs LLC, 1225 Northmeadow Parkway, Suite 104, Roswell, GA 30076, USA

## Abstract

Placental tissues encompass all the tissues which support fetal development, including the placenta, placental membrane, umbilical cord, and amniotic fluid. Since the 1990s there has been renewed interest in the use of these tissues as a raw material for regenerative medicine applications. Placental tissues have been extensively studied for their potential contribution to tissue repair applications. Studies have attributed their efficacy in augmenting the healing process to the extracellular matrix scaffolds rich in collagens, glycosaminoglycans, and proteoglycans, as well as the presence of cytokines within the tissues that have been shown to stimulate re-epithelialization, promote angiogenesis, and aid in the reduction of inflammation and scarring. The compositions and properties of all birth tissues give them the potential to be valuable biomaterials for the development of new regenerative therapies. Herein, the development and compositions of each of these tissues are reviewed, with focus on the structural and signaling components that are relevant to medical applications. This review also explores current configurations and recent innovations in the use of placental tissues as biomaterials in regenerative medicine.

## 1. Introduction

**Figure 1:**
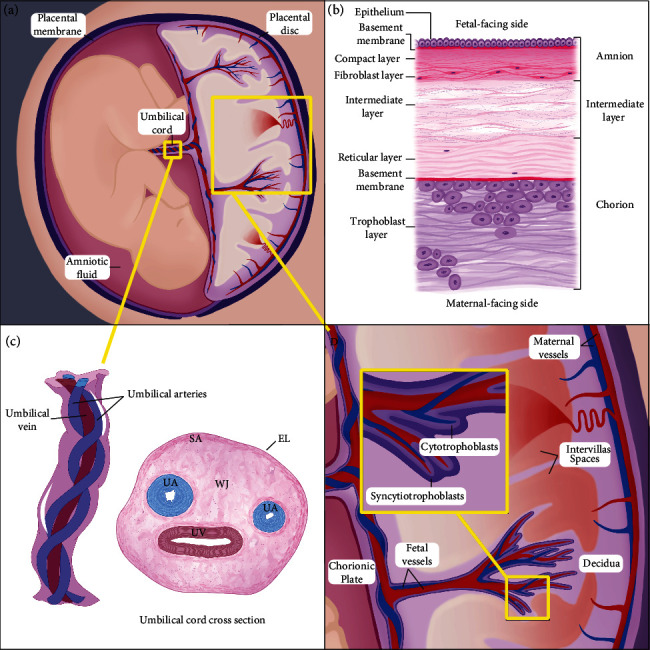
The anatomy of placental tissues. (a) The anatomy of the fetal compartment, (b) the anatomy of the placental membrane layers, (c) the anatomy and cross section of the umbilical cord, including the epithelial layer (EL), subamnion (SA), Wharton's Jelly (WJ), umbilical arteries (UA), and umbilical vein (UV), and (d) the anatomy of the placental disc.

Placental tissues, or extra-embryonic tissues, also commonly referred to as birth tissues, support and protect fetal development during gestation. They include the placental disc, the umbilical cord, placental membrane, and amniotic fluid (Figure 1A). These tissues are transient, as they only exist to support the fetus until birth. Placental tissues are of great interest in medicine, specifically as a biomaterial because of the exceptional properties of fetal tissues and the ease of access to them as raw materials [1, 2]. Unlike cadaveric tissue, placental tissues are of a consistent age, donated from a screened population of healthy donors, do not require extensive procedures to collect, and would otherwise be discarded as medical waste [3]. Common tissue collection practices involve packaging for transport following routine cesarian section deliveries, and vaginal deliveries under certain circumstances, with sterile materials and transport solutions. Sterile containers are commonly used to transport the tissue to avoid contamination, and storage time is kept as minimal as possible under refrigerated conditions to ensure material stability [3, 4].

In the 16^th^ century, medical use of the human placenta was described in China [5]. The first documented use of placental tissues in modern medicine dates back to the beginning of the 20^th^ century where application of placental membrane in skin grafting was first published showing superior efficacy over cadaveric tissue by Davis in 1910 [6]. The first studies showing therapeutic efficacy of placental tissues appear between the 1930s and 1980s [2]. Several reports were published in the 1930-1940s on the use of placental membrane in surgical and wound applications followed by clinical reports and trials through the 1970s and 1980s. As contemporary standards of cleanliness and sterility developed through the ‘80s and ‘90s, scientists began to experiment with dehydration and sterilization of the tissue. This led to the increased safety, usability, and access of today's shelf-stable products.

Given the clinical benefits of placental membrane grafts in wound and surgical applications, research has further focused on developing and testing new tissue- and cell-based therapies derived from placental tissues [4]. Since the turn of the century, applications of these tissues have included uses in cell therapy, bioengineering, and regenerative medicine [3]. The majority of clinical studies have utilized placental membrane, umbilical cord veins, and amniotic fluid [3]. However, extensive preclinical experimentation has been performed on the placental disc, Wharton's Jelly, and umbilical arteries [3]. The wide array of applications stems from the varied functions and forms of the placental tissues and their rich composition of extracellular matrix (ECM) components, signaling molecules, and growth factors [3].

Over the last 30 years there has been vast interest in the use of implanted, injected, or cultured stem cells as a therapy to help regenerate damaged tissues [7]. Placental tissues are a rich source of stem cells that have been extensively researched. The use of multipotent mesenchymal stromal cells (MSCs) from umbilical cord blood, Wharton's Jelly, and placenta, for instance, have been thoroughly described [8, 9] and are not a subject of this review. Recent research regarding the regenerative mechanism of action of MSCs has shown that they provide therapeutic benefit through the release of cytokines [10]. This, along with the clinical success of non-viable placental tissue allografts, further suggests a therapeutic potential for placental tissues as biomaterials [2]. This review will discuss the raw material potential of placental tissues as sources of ECM components, scaffolds, signaling molecules and growth factors for use in regenerative therapies. We will emphasize the structural and soluble biochemical composition of these tissues and discuss current innovations using these tissues as biomaterials in regenerative medicine.

## 2. Placental Membrane

### 2.1. Anatomy and Development

The placental membrane (PM) surrounds the developing fetus and creates the amniotic fluid-filled fetal compartment during gestation (Figure 1A). The PM is structured in three major layers: the fetal-facing amnion, the maternal-facing chorion, and the intermediate layer in between, as shown in Figure 1B [11, 12]. The amnion, which is composed of epithelium, basement membrane, compact layer, and fibroblast layer, encloses the fetus and the amniotic fluid, which cushions and protects the fetus during gestation [13]. The chorion, the maternal-facing layer, in contact with the maternal decidua, is comprised of three layers: the reticular layer, a basement membrane and a trophoblast layer [11, 14, 15]. The chorion forms a barrier between the fetal environment and the maternal immune system [16, 17]. The intermediate layer is a spongey network of collagens and proteoglycans that functions as a physical barrier between the amnion and chorion. The structure of this layer aids in the movement of the amnion along the chorion, supported by the high concentration of hyaluronic acid (HA) which hydrates and lubricates the tissue [11, 18–20].

During gestation, the amnion and chorion form separately after blastocyst implantation around day 14 of gestation, alongside the development of the placental disc [21, 22]. The wall of the blastocyst becomes the chorion. By day 10 post-conception, the amnion is visible, forming from the amniogenic cells that make up the inner trophoblast layer of the cellular mass of the embryo [21]. The PM continuously grows throughout gestation to accommodate the growing fetus. Following the end of month 3 of gestation, the amnion is visibly separated from the chorion leave, the precursor to the chorion, by the chorionic cavity. As the amniotic sac fills with fluid and gradually expands, the amnion adheres to the innermost surface of the chorion and the chorionic cavity disappears. Although now closely adhered, amniotic and chorionic layers of the PM do not fuse and remain histologically separate [21]. At term, the literature reports native PM thickness can range from 0.02 mm to 0.6 mm and the average surface area of the PM is 1600 cm^2^ [1, 23].

### 2.2. Physiology

The PM acts to provide a barrier and protect the fetus from the maternal environment and withstand the stretching and pressure created by the fetus as it develops and moves [14, 24]. In addition, the PM is metabolically active and provides an immune barrier to infection and a barrier to immune rejection by the mother [25]. Over the course of gestation, the PM constantly undergoes matrix remodeling to maintain its barrier integrity and accommodate a growing fetus [26]. The composition of each layer of the PM varies slightly to support the overall function of the membrane. The basement membrane of the amnion is a dense tissue layer that acts as a permeable barrier for the exchange of nutrients [11, 27]. The compact layer provides tensile strength to the membrane and is the most fibrous of the amnion layers due to the high content of collagens secreted from the mesenchymal cells in the fibroblast layer [11, 12, 28]. The fibroblast layer contributes to the tensile strength of the membrane and provides a scaffold for cell-cell interactions [29, 30]. The intermediate layer functions as a barrier between the amnion and the chorion, providing mechanical support in the form of a nonfibrillar network made up mostly of proteoglycans, glycosaminoglycans (GAGs) and type III collagen [11, 18, 20]. The reticular layer provides a scaffold for the chorion with a network of fibers and is embedded with mesenchymal cells similar to the fibroblast layer in the amnion [31]. The basement membrane between the reticular layer and the trophoblasts increases the structural integrity of the chorion by providing cell scaffolding for the trophoblast layer and contributes to the immune privilege of the tissue [16, 32, 33]. The trophoblast layer, the outermost layer of the chorion, consists of trophoblasts, myofibroblasts and macrophages [16].

The overall PM structure supports resident cells while maintaining tensile strength and elasticity during the regular turnover of the ECM that is facilitated by endogenous cytokines in the PM [34]. The PM is an immune privileged tissue contributing to its function as an immune barrier between the mother and fetus, preventing maternal rejection of the fetus. Studies in the early 1980s found no human leukocyte antigen (HLA) -A, -B, -C, or -DR molecules in human amnion, but more recent studies have detected these class I and II molecules in various cells of the tissue [35]. However, while almost all cells of the PM have been shown to express class I HLA molecules, the tissue has been found to be immunologically inert with almost no reports of immune rejection [35, 36] and there is little to no major histocompatibility complex (MHC) reactivity in unmatched adult recipients [36]. This limited reactivity is reportedly due to the active suppression of B cells, T cells, and dendritic cells, from interactions such as the binding of CD8+ T cells to HLA-G molecules [35, 36]. Further Fas ligand expression on amnion epithelial cells is suggested to signal apoptosis of host lymphocytes, helping to prevent maternal rejection of the fetus, along with the immunosuppressive actions of cell surface and soluble HLA-G [35].

### 2.3. Structural Composition

The PM is composed of a network of collagens (types I, III, IV, V and VI), laminin, fibronectin, HA, vitronectin, elastin, and proteoglycans, which provide mechanical strength, elasticity, flexibility, and appropriate stiffness to support the barrier integrity in the uterine environment (Table 1) [12, 18, 34, 37, 38]. Each layer of the PM has a different composition to serve its individual function [20]. The ECM of the amnion contains collagens types I, III, IV, V and VI secreted by the mesenchymal cells in the fibroblast layer, as well as elastin, fibronectin, laminin, HA, and sulfated proteoglycans [12, 19, 34]. The chorion ECM consists of collagens types I, III, IV, V, and VI, elastin, fibronectin, laminin and HA [34]. The intermediate layer is composed of collagen types I, III, and IV, sulfated proteoglycans and glycoproteins. Due to the high concentration of proteoglycans and glycoproteins, the intermediate layer allows the amnion and chorion to glide against each other [14, 18, 28].

The PM contains different types of collagen (types I, III, IV, V, and VI) secreted by fibroblasts which serve different purposes depending on which layer they are found in [28]. Collagen types I and III form parallel bundles throughout all PM layers, except the trophoblast layer, to support the mechanical integrity of the tissue during gestation [20, 28]. Collagens type IV and V form connections to the basement membranes of both the amnion and the chorion [39]. The fibrous structure of the amnion is attributed to the collagens type I, III, V and VI present [20]. The chorion, the thickest of the layers, contains large amounts of collagens type I and IV, which form scaffolding for resident cells in the PM [20, 28]. Additionally, the presence of collagen in the trophoblast layer of the chorion assists in the anchoring of the PM to the maternal decidua [14, 20]. The intermediate layer is comprised of collagen type I, III and IV and contains a higher density of proteoglycans and mucopolysaccharides [20]. PM is attractive to researchers interested in collagen-rich sources for wound healing applications because of the role of collagen in reducing inflammation in wound healing [40].

Glycoproteins, such as fibronectin and laminin, are ECM components that contribute to the structural integrity of the PM. Fibronectin interacts with collagens and proteoglycans, and influences cell morphology, adhesion, migration, proliferation and differentiation by binding to the cells in both the amnion and chorion [28, 41] Laminins provide a connection between neighboring membrane layers by binding to other proteins, like collagens [28, 42], [43]. Proteoglycans contribute to the tensile strength and integrity of the PM, as well as to cell proliferation and differentiation, and assist in binding of growth factors in the other layers of the PM [14, 18]. When utilized as a biomaterial in wound management, this naturally occurring ECM scaffold can provide structural support and facilitate intercellular and intracellular signaling between cells, cell attachment and cellular migration essential to proper wound remodeling [44, 45]. PM ECM components have been shown to contribute to decreased inflammation, prevention of infection, and reduction in scarring and pain at the site of injury [44, 46].

The ECM also contains components that give the PM flexibility and cushioning from the pressures of gestation. Elastin fibers contribute to the integrity and tensile strength of the PM as it stretches during gestation [15, 20]. GAGs, such as HA, are another component that provides the ECM with elasticity and lubrication. Studies on the distribution of HA in PM have observed its presence in collagen-rich (specifically types IV and V) areas of the tissue, for example, the basement membrane or reticular layer of PM, as well as intermediate layer, but not in the epithelium of the amnion. The chorion and intermediate layer have a greater presence of HA than the amnion [34, 47, 48]. The GAG content of the intermediate layer, specifically the HA, contributes to the hydrophilic nature of the layer [20]. HA has also been shown to play a role in the reduction of inflammation and readily accessible biomaterials that provide HA are of growing interest in the regenerative medicine field [20, 38].

### 2.4. Cytokines & Antimicrobial Components

Extensive research into the cytokine content of both native PM and processed PM allografts has been done to help understand the role of PM in gestation and potential clinical applications. The layers of the PM are a reservoir of cytokines and growth factors which regulate the pathways that maintain the ECM structural integrity during gestation (Table 2). Evaluating the cytokine and growth factor content in the amnion, the intermediate layer, and chorion is of interest for varying applications of the tissue [20, 49–51]. Specific focus has been given to growth factors such as epithelial growth factor (EGF), fibroblast growth factors (FGFs), vascular endothelial growth factor (VEGF), transforming growth factor alpha (TGF*α*), transforming growth factor beta (TGF*β*), keratinocyte growth factor (KGF), hepatocyte growth factor (HGF), and platelet-derived growth factors (PDGFs), interleukins (ILs) such as IL-4, IL-6, IL-8, and IL-10, interferons (IFNs), and protease inhibitors including tissue inhibitors of metalloproteinases (TIMPs) TIMP-1, TIMP-2, and TIMP-4 because of their applicability to therapeutic applications [49, 50, 52–54]. These signaling components help regulate multiple aspects of the healing cascade, including angiogenesis, inflammation, and tissue remodeling [20, 51]. Basic FGF (bFGF), endocrine-derived vascular endothelial growth factor (EG-VEGF), VEGF and PDGF-AA, and PDGF-BB stimulate the activity and migration of matrix metalloproteinases (MMPs), which are responsible for the breakdown of existing ECM in preparation for remodeling of the PM [34, 55]. TIMPs are important inhibitors that regulate the activity of MMPs to prevent excessive breakdown of the ECM and deposit ECM components [56, 57]. In addition to the active role MMPs play in the remodeling of the placental membrane during gestation, they are an active component in the onset of labor, where high levels of MMPs and reduced levels of TIMPs induce the degradation of the PM ECM resulting in membrane rupture [55]. Specifically, at the onset of labor, MMP-9 concentrations are mainly localized near the cervix and the concentrations rise throughout the PM as labor progresses [55].The tensile strength of healthy term PM has been correlated to the distribution of MMP-9 with the weakest areas having the highest concentration [31].

The amnion and chorion are comparable in terms of the types of cytokines present but differ in their distribution and concentration [51]. The concentrations range from less than 1 pg/mg to over 6,000 pg/mg, normalized by dry weight, depending on the thickness and location [33, 38, 50, 58]. Multiple cytokines have been found in significantly higher concentrations in fresh chorion compared to other layers, including adiponectin (APN), angiogenin (ANG), ANG-2, bFGF, EG-VEGF, HGF, insulin-like growth factor 1 (IGF-1), PDGF-AA, PDGF-BB, TIMP-2 and TIMP-4 [51]. The greater concentrations per surface area found in the chorion are attributed to the chorion being thicker than the amnion [51]. The amnion layer, however, does contain higher levels of several components, including galectin-7, IL-1F5 and TGF-*β*1 [51]. The proteomic profile of the intermediate layer has been recently investigated, demonstrating that the layer not only contributes to the overall structural integrity of the membrane but also functions as a source of signaling components important to wound healing applications, such as PDGF-AA, PDGF-BB, TIMP-1, TIMP-2, TIMP-4, TGF-*β*1, bFGF, EGF, and VEGF [20].

The PM also serves as a barrier to bacterial infection during gestation, containing layers of antimicrobial peptides, such as human defensins, elafin, secretory leukocyte protease inhibitor (SLPI), and histones H2A and H2B [59, 60]. Human *β*-defensin (HBD) is produced by epithelial cells in the PM [61–63]. Defensins are antimicrobial peptides that prevent the growth of bacteria [61]. The PM also contains cystatin E which has anti-viral properties [64]. There are greater levels of bacterial inhibition from the maternal-facing chorion than the fetal-facing amnion, which points to the role of the chorion as a key barrier layer in preventing infection and rejection [63].

### 2.5. Clinical Applications and Uses in Regenerative Medicine

The PM has the longest history of use in modern medicine of all placental tissues with the first documented case in 1910 where it was used in skin grafting [6]. This membrane has been extensively researched for use in wound applications, where the sheet configuration easily translates to a wound covering. Apart from its regenerative properties, the tissue is attractive in wound healing applications because it is a sustainable material, typically classified as medical waste, that is immune privileged [36, 65]. While acute and chronic wounds and burns are still the most prevalent application for PM, there is a wide variety of surgical and sports medicine applications where PM is being investigated as a treatment, including ocular reconstruction, spinal surgery, nerve reconstruction, dermatological cases, and as a barrier membrane for adhesion or scarring prevention [4, 11, 12, 36, 66].

The wide array of applications and advances in tissue processing technology has led to the emergence and investigation of numerous PM configurations, including as single-layer, multi-layer, full-thickness and composite grafts, as a micronized powder, and as an injectable matrix [11, 34, 67–70]. Processing and preservation techniques also vary. Depending upon the application, the PM may be left untreated or decellularized to remove donor cells and DNA from the tissue, for example using dispase, EDTA, etc.[71, 72]. Additional processing techniques can include dehydration methods such as oven dehydration and lyophilization, cryopreservation, and cryomilling [26, 67, 69, 70]. Studies have shown that lyophilization prevents deterioration at room temperature for a more stable product that is comparable to fresh placental tissue [66, 69, 73]. Characterization of placental membrane configurations has demonstrated retention of their regenerative potential after processing. However, some studies have reported reduced concentrations of cytokines, as well as altered tissue structures and appearance compared to fresh PM [20, 37, 51, 58].

Over the last two decades, there has been an increased use of PM allografts in the treatment of wounds and burns as more evidence continues to emerge regarding the ECM, therapeutic components, and proven efficacy [20, 74, 75]. Processed PM has been a successful treatment for venous leg ulcers (VLUs), diabetic foot ulcers (DFUs), and chronic wounds, demonstrating decreased time to wound closure [74], On a cellular level, the PM ECM has scaffold characteristics that are beneficial for anchorage-dependent cell attachment and endogenous cytokines that promote proliferation, angiogenesis, cellular migration, tissue remodeling, and create an environment for the resolution of the inflammatory stage of healing and improved remodeling [12, 20, 21, 34, 37, 38]. Clinical applications of dehydrated PM as burn dressings have shown that the PM encourages re-epithelialization of damaged tissue, stimulated by EGF, KGF and HGF, reducing inflammation, decreasing healing time and decreasing scar tissue development [76]. Implanted acellular PM graft in a subcutaneous mouse model encouraged migration and infiltration of host cells during the healing process, specifically of fibroblast-like cells, macrophages, endothelial cells, and fewer T-cells than the control graft [77]. PM has been successfully used in ophthalmology for almost 30 years for the treatment of corneal lesions, retinal detachment, and limbal cell regeneration [78, 79]. Both intact and denuded PM used in ocular reconstruction in a rabbit model achieved re-epithelialization and integration into the rabbit tissue by 3-weeks post-surgery, with lower inflammatory responses noted in denuded PM treated eyes [80]. Extract from digested amnion used in eye drops for corneal treatment has been shown to successfully cultivate limbal stem cells clinically [81].

Placental membrane-derived materials have been increasingly used surgically in reconstructive, vascular, OB-GYN, abdominal, spinal, and neurological cases. Dehydrated PM usage has been shown to result in superior recovery and lower rates of reherniation following microdiscectomy [82]. Retrospective case studies have shown the potential benefits of PM to prevent retethering of the spinal cord following microsurgical intradural lysis adhesion [83]. The preventative use of PM against failed back surgery syndrome (FBSS) caused by epidural adhesions resulted in a significant decrease in the incidence of epidural fibrosis formation compared to standard closure in canine and rat models [84, 85]. There is evidence of PM use in a variety of reconstructive applications including vaginoplasty and vestibuloplasty [86, 87]. The clinical uses of PM in the field of urology are numerous, including penile reconstruction, microsurgical cord denervations, posterior urethroplasty, hypospadias repairs, and urogenital indications, such as vesicovaginal fistulas, as reviewed by Ootamasathien et al. (2017).

A review of the literature will show dozens of animal study investigations of PM as an intraabdominal adhesion barrier, resulting in improved outcomes in various animal models. A retrospective cohort study of 120 patients with 40 patients receiving dehydrated PM barriers used following intrauterine adhesiolysis and 80 patients in the control group found PM to reduce adhesion reformation and improve subsequent menstruation compared to the control group [89]. A study investigating the use of PM as an adhesion barrier following laparoscopic resection of endometriosis with adhesiolysis found that after 1-2weeks no adhesions were observed at point of PM placement in 14 out of 15 cases [90] In a rat model, PM used as a nerve wrapping resulted in a decrease in scar tissue development and an increase in sciatic function, suggesting that in addition to functioning as a barrier, the PM assisted in regenerating the nerve [91]. Recently, a reconstituted, injectable PM powder was investigated in a post-myocardial infarction tissue repair procedure in a rat model, resulting in a decrease of myocardial infarction scar formation and improved left ventricular function [68].

More recently, use of PM in the field of dermatology has been explored, including applications of PM in Mohs surgery, in the management of chronic radiation necrosis, and in the treatment of pyoderma gangrenosum cases [92–94], A case series of the use of dehydrated PM Mohs micrographic surgery for large full-thickness (to the bone) scalp wounds found treatment was well tolerated and resulted in the development of granulation tissue [95]. A case of a 77-year old female with pyoderma gangrenosum treated with dehydrated PM as an adjunct to immunosuppressives reported pain reduction from 10/10 to 5/10 within hours of application that decreased to 0/10 after 1 week and a 56% reduction in wound size after 3 applications.[93].

PM products have also been investigated and used in the past decade to treat various musculoskeletal and orthopedic conditions, including ligament and tendon repair and osteoarthritis (OA) [79]. In a case study focused on flexor tendon injuries, a blunt dissected amnion wrapped tendon resulted in a higher range of active motion, a higher rank on the Strickland and Glogovac scale, compared to the control group [96]. Similarly, a combination injectable matrix composed of PM and umbilical cord has been used to treat knee OA in humans. Results from the pilot study showed an increase in cartilage thickness/volume and a decrease in lesions compared to the control group [97]. In a rat model for Achilles tendon repair utilizing type II diabetic rats to impair healing, dehydrated dual layer amnion/chorion showed improved cell migration, lower failure rates, and more favorable mechanical strength of the tendon compared to untreated controls [98]. Micronized PM has been utilized as an injectable treatment for OA, and in rats with induced OA has shown to result in joints with higher proteoglycan content than controls [99]. Intra-articular injection of lyophilized PM in rabbits for OA showed similar improvements compared to a saline control [79]. A controlled, multi-center trial for PM-derived barriers to encase flexor tendons following repair resulted in significantly increased range of motion and functional scores compared to control [79].

## 3. Amniotic Fluid

### 3.1. Anatomy and Development

Amniotic fluid (AF) begins to form around week two of gestation and is the product of water accumulated from maternal circulation that has filled the extracelomic cavity (Figure 1A). In addition to water accumulation via maternal vessels of the decidua and chorion laeve, AF constituents are derived from filtration from the fetal vessels of the umbilical cord and chorionic plate, secretory processes of the amniotic epithelium, and filtration from intracorporeal fetal vessels via the fetal skin [100]. As pregnancy progresses to week 8, prior to the keratinization of fetal skin, water, proteins and other metabolites diffuse freely and bidirectionally between the fetus and AF [101–103]. During this time, the composition of AF resembles maternal and fetal serum. To meet the requirements of development, towards the second half of gestation, the composition of AF begins to change due to fluid regulation and exchange pathways, such as fetal swallowing, lung fluid excretion, fetal urination, and intramembranous absorption [104]. AF volume is primarily regulated through fetal swallowing and intramembranous exchange across the PM. This intramembranous flow transfers solutes and fluid between the maternal circulation and the amniotic cavity. Meanwhile, fetal swallowing allows these substances to be transferred from the amniotic cavity into fetal circulation [104].

A low density population of heterogeneous AF cells called amniocytes is present in AF that are derived from the fetal tissue [105]. Reported cell concentrations in freshly collected AF samples are on the order of magnitude of 10^5^ cells/mL [106, 107]. Amniocytes collected from routine amniocentesis performed during 14-16 weeks gestation have been differentiated and subclassified into one of 3 main groups based on their morphological and growth characteristics in culture: 60.8% amniotic fluid specific type (AF-type) cells, 33.7% epithelioid (E-type), and 5.5% fibroblastic type (F-type) [108]. To identify subclassification of amniocytes in AF, cell culture must be performed to observe the morphological characteristics of the matured colonies [108]. Morphologically, mature E-type cells are large polygonal cells with smooth margins that grow in close contact with each other in culture. They resemble AF-type cells but can be distinguished by their broader growth margin [108]. Mature AF-type cell colonies have pleomorphic morphology and can be differentiated after 12-14 days. They are distinguished by their characteristic “bulls-eye” growth pattern that is not found in E- or F-type AF cell colonies [108, 109]. It has been suggested that AF-type cells in AF fluid are derived from extraembryonic, fetal trophoblastic tissues, yet their function during gestation is not well understood [110, 111]. F-type cells originate from fetal fibrous connective tissue and dermal fibroblasts and generate a ribbed and streaming pattern as they mature in culture. They are spindle-shaped and overlap, forming multi-layered sheets in areas of greater density when cultured [108, 109]. A very small subpopulation, approximately 0.1-0.5%, of AF cells are CD117+ and have differentiation potential. AF mesenchymal stem cells (AF-MSCs) have been an area of focus for regenerative medicine in recent years [112–115]. As the vast majority of studies focused on amniocytes have been performed in vitro, our understanding of their proliferative potential and heterogenous phenotype in vivo is still evolving, especially given the processing of AF-derived products that would likely render MSCs non-viable [112, 116, 117]. A 2019 study found that while 3 AF-derived injectable products all contained signaling components that modulate healing, there were no cells with known characteristics of MSCs in the product [117]

### 3.2. Physiology

One of the most important functions of AF is its role in fetal support and protection throughout gestation [103]. While AF provides a unique environment that supports fetal growth and movement, it plays a large role in cushioning and protecting the fetus against thermal and mechanical shock [103]. AF also acts to help protect the integrity of the umbilical cord by providing a mechanical cushion between the fetus and the uterine wall, preventing the umbilical cord from being compressed during gestation [101]. Separate from mechanical protection, AF contributes to the innate immunity of the fetal system. A host of antimicrobial and bacteriostatic peptides have been identified in AF (Table 2), which help protect the fetal environment from infection or common bacterial or fungal pathogens [103].

Additionally, AF acts as a reservoir for nutrients, such as essential free amino acids, proteins, lipids, electrolytes, and carbohydrates, as observed in mid-trimester AF [118]. These nutrients originate from maternal plasma and the placenta and are transported to the fetal cavity through placental circulation or, less commonly, across the PM and into the AF through carrier-mediated transporters and channels located in the PM [104, 119, 120]. The composition of these nutrients constantly changes to meet the needs of the developing fetus [118]. At full-term, AF composition is about 98% water and electrolytes. Peptides, carbohydrates, lipids, hormones, and signaling molecules comprise the other 2% [101]. These non-water constituents are soluble components that aid in the structure, protection, nutrition, and overall well-being of fetal development. Glucose is the primary energy source for the fetus during gestation and is transported to the fetus via the placenta as well as from swallowing AF [121, 122]. In an animal model, essential amino acids in AF, such as glutamine, are required for gastrointestinal development, growth, and function, as well as fetal nitrogen and carbon metabolism. Amino acid concentrations naturally decrease towards the end of gestation but could be further reduced by alterations in maternal diet [120].

### 3.3. Structural Composition

AF analyzed mid-trimester contains ECM components that are secreted by fetal cells suspended in the AF or shed from placental tissues and deposited into AF by fetal urine (Table 1) [123, 124]. Collagen types I, III, and IV are major components of the PM [125]. As the PM is weakened in preparation for spontaneous rupture of the fetal membranes, enzymatic constituents of AF, such as MMP-9, degrade and metabolize the collagen present in the PM, likely leading to collagen deposition into AF at term [125, 126].

It has also been proposed that cultured fetal F-type cells in AF are a dictator of collagen deposition, the main component of the fetal dermal ECM [108], and are responsible for the production of type I and type III collagen and fibronectin in fetal ECM development [109, 111, 127]. In culture, F-type and AF-type amniocytes produce a spectrum of collagenous proteins. In vitro F-type cells predominantly synthesize type I and type III collagen, but AF-type cells synthesize considerably less collagen [128]. Although the importance of these amniocytes during gestation is not well studied, these findings suggest a role of amniocytes in collagen synthesis [127], which may contribute to signaling fetal wound repair and dermal growth [109, 129]. The function of E-type cells in AF is not widely understood but they have been shown to be involved in secretion of type IV collagen and cell-to-cell organization of both keratin and vimentin fibers in culture, further suggesting their role in fetal development [108, 130].

AF contains multiple GAGs and nutrients that may play an important role in providing protection, cushioning, and lubrication to the developing fetus [131–133]. The cushioning and lubrication properties of ECM components present in AF, such as HA, have been a topic of interest in regenerative medicine for their potential therapeutic effects on treating synovial joint pain and prevention of adhesion formation [47, 134]. HA (34%), chondroitin-6-sulfate (20%), non-sulfated chondroitin (14%), chondroitin-4-sulfate (13%), heparan sulfate (6%), and dermatan sulfate (5%) make up most of the GAGs found in AF [135].

The relatively low concentrations of hyaluronidase in AF allow for an environment rich in HA [136]. HA serves to increase AF viscosity, provides support and lubrication in the uterine environment, and may also play an important role in fetal wound healing [137]. Studies show that HA provides a mesenchymal signal for regenerative healing in fetal wounds, contributing to the scarless healing observed in fetuses, and potentially providing intercommunication between the fetus and surrounding tissues through the AF [132, 138, 139].

Fibronectin is a glycoprotein found in high concentrations in AF compared to plasma [140]. It is secreted by both F-type and AF-type cells and is associated with the pericellular and PM ECM [127]. The physiological functions of fibronectin in AF are relatively unknown, but fibronectin present in AF carries double the carbohydrates when compared to plasma fibronectin, allowing for better protection against proteolytic digestion [140]. Fibronectin may be responsible for the rapid, scarless wound healing observed in fetal wounds as deposition of fibronectin occurs earlier in fetal wounds than in adult wounds [131, 141].

### 3.4. Cytokines and Antimicrobial Components

AF is rich in cytokines that are secreted rapidly from fetal cells, including fetal macrophages, suspended in AF as they are signaled to aid in tissue remodeling, protease inhibition, antimicrobial defense, and angiogenic and anti-inflammatory signaling during gestation (Table 2) [103, 114, 142]. Many are investigated for their role in therapeutic applications, such as EGF, bFGF, IL-1ra, PDGF-BB, VEGF, and TIMPs. It is believed that many of these cytokines do not readily cross the placenta. Interleukins present in AF are important for cellular communication and the regulation of inflammation [143–145]. Most research on the proteomic make up of AF comes from pre-term analysis via amniocentesis in the study of biomarkers indicative of spontaneous pre-term delivery and other conditions, such as pre-eclampsia [16]. Further studies have investigated the AF-MSC secretory profile and their impacts on wound healing, revealing that AF-MSC conditioned media has higher profiles of IL-6, IL-8, VEGF, EGF, and TGF-*β* that successfully promoted healing pathways in dermal fibroblasts [146]. In second trimester AF, the two most abundant cytokines are anti-inflammatory IL-1ra and pro-inflammatory interferon gamma-induced protein 10 (IP-10) [142]. IL-1ra in AF helps protect the fetus against inflammatory damage caused by overactivation of IL-1*β* [142]. IGF-1 is an important factor in tendon healing, as it has a positive effect on tenocyte proliferation in vitro [147]. In AF, growth factors such as IGF-1 are primarily involved in intrauterine growth via enterocyte proliferation and has been shown to increase fetal intestinal growth and maturity [148, 149]. The IGF system is the dominant endocrine determinant of fetal growth because of its role in regulating insulin and growth hormone [150]. IGF-1 plays an important role in tissue regeneration and wound healing, and has been especially studied in the repair of tendon and joints [151, 152]. For instance, IGF-1 has been shown to have a positive effect on tenocyte proliferation in vitro, and to play a role in preventing cartilage metabolism in the development of OA [147, 151].

AF is also a key regulator of the innate immune system and contains enzymes, immunomodulatory mediators, and antimicrobial polypeptides and lipids that aid in antimicrobial defense and fetal skin immunity [136]. Human *β*-defensin-1 (HBD-1) concentrations increase in the presence of microorganism infection in the amniotic cavity, demonstrating the participation in host-defense mechanisms [153]. Other critical antimicrobial components of AF include HBD-2, lysozyme, cystatin C, and lactoferrin, which prevent infection via iron binding function that inhibits bacterial, viral, fungal, and parasitic infection.

### 3.5. Clinical Applications and Uses in Regenerative Medicine

AF has become increasingly used in regenerative medicine applications because of the rich mixture of growth factors, antimicrobial factors, amniocytes, and GAGs [154]. The material translates well as a raw material for medical use because of its availability and the immune privileged state of placental-derived tissues. The antimicrobial constituents of AF contribute to its favorable potential as a biomaterial in regenerative medicine, especially in the treatment of chronic wound infections [143, 154]. Many configurations and processing techniques for AF have been investigated for therapeutic applications. Minimally filtered or centrifuged AF solutions have been used or investigated in near-native states for the treatment of OA, tendonitis, plantar fasciitis, wound healing, and adhesion prevention [155–159]. Unlike most allograft or biological tissues currently used for biomedical purposes, AF is advantageous for the supplementation of fluid environments. Following filtration or centrifugation to remove various constituents, different preservation techniques have been investigated, including cryopreservation and lyophilization to a powder; additionally, combinations of AF-derived allografts and other materials, such as alginate-based hydrogels have been investigated [137, 154, 157, 160, 161]. AF has also been used to make amniotic suspension injectable products, containing a combination of micronized human PM and AF components, such as cells [147, 156, 158].

AF-derived materials have been investigated for use in treating OA, rheumatoid arthritis (RA), various tendon injuries, bone defects, corneal defects, abdominal adhesions, and in novel wound healing therapies [133, 154]. As an injectable therapy, AF has been administered in the form of an amniotic fluid suspension or as a fresh medium [147, 156, 158, 162]. It has been suggested that the high HA content in AF makes it a promising candidate for treatment of RA and OA when injected into the synovial fluid of diseased joints [162]. This is likely due to the cushioning and protection properties of HA and the anti-inflammatory components of AF [47, 156, 162]. Vines et al. found that a single intra-articular injection of an amniotic suspension allograft (ASA) containing cryopreserved amniotic fluid cells statistically increased IgG and IgE levels in moderate to severe OA patients, demonstrating the feasibility for the treatment of knee OA [158]. In an animal model with induced OA, treatment with cryopreserved ASA containing AF cells resulted in significant decreases in pain thresholds and swelling [156]. There were also increased levels of IL-10 in the synovial fluid surrounding the joint induced with OA, suggesting the promotion of an anti-inflammatory environment when treated with the ASA-containing AF cells [156]. When studying the effect of ASAs containing AF cells on tendon healing, Kimmerling et al. found that ASA injections greatly increased density and migration of tenocytes as well as deposition of ECM proteins, including collagen and elastin, in vitro [147]. In the presence of the ASA, less TGF-*β*1 was produced and IL-8 was downregulated, suggesting an anti-inflammatory effect of the AF-containing allograft. These findings allow for a better understanding of the potential use of ASAs containing AF cells in tendon repair and healing [147]. AF injections have also been investigated for stimulating neochondrogenesis when injected under free perichondral grafts in rabbits. A single 0.3mL dose of freshly collected AF was effective in promoting proliferation and differentiation of chondrocytes as well as stimulating new cartilage formation in the treatment of certain cartilage defects [162]. It was suggested that these results were likely due to the high concentrations of HA and other growth factors in AF [162]. In an animal model, freshly collected AF was applied topically to tendon sheaths to evaluate the effect on peritendinous adhesion formation and differences in tensile strength. Significantly fewer adhesions were observed in tendons treated with AF and they had significantly higher load values compared to the untreated tendon controls. Their results suggest that human AF is effective in facilitating tendon healing and preventing adhesion formation [134].

Amniotic fluid for the treatment of chronic soft tissue defects and wounds has been increasingly utilized over the last decade [154]. In chronic diabetic wounds, AF is a relatively new innovation in the treatment of chronic soft tissue defects and wounds [154]. In chronic diabetic wounds treated with processed, lyophilized AF, Bazrafshan et al. found significant increases in the rate of wound closure compared to control wounds not treated with AF [154]. A significant increase in the number of large vessels in the wounds injected with AF was also observed. Recently, alginate hydrogel-electrospun silk fibroin fibers combined with AF resulted in increased cellular proliferation, spreading, and higher collagen secretion in vitro [161].

Longaker et al. found that in incisional wounds of fetal lambs, cultured fetal fibroblasts in AF produced more collagen than adult fibroblasts, leading to a faster rate of collagen diffusion into the AF from the ECM of placental tissues, and therefore reduced scar formation [163]. In an in vitro wound model, Nyman et al. found that the rate of re-epithelialization was significantly increased and hyaluronidase activity was inhibited when treated with, centrifuged, filtered mid-trimester AF collected from amniocentesis [137]. Fresh AF has been used to treat various corneal disorders because of its reported positive effects on corneal sensitivity and decreased scar formation in the cornea [160]. In a study evaluating the effect of fresh AF on corneal epithelial defects in rabbits, topical application of AF to the cornea resulted in accelerated re-epithelialization and corneal wound healing and protection of corneal keratinocytes [160]. EGF is important in AF for promoting the growth of various developing organ systems, importantly the gastrointestinal tract and the lungs [164]. Investigation of EGF stimulation of hyaluronan synthesis in vitro resulted in abundant hyaluronan levels during keratinocyte migration into an open wound [165]. Signaling components from AF have been shown to retain their bioactivity after processing and lyophilization, promoting increased wound closure rates, cellular proliferation, and stimulating angiogenesis in induced chronic diabetic wounds [154].

## 4. Umbilical Cord

### 4.1. Anatomy and Development

The umbilical cord (UC) is a tubular connection between the fetus and placenta and acts as a conduit for vessels that provide an exchange of oxygen and nutrient-rich blood between the developing fetus and the placenta [166]. The UC protects and facilitates blood flow despite various forces within the fetal compartment that could compress, kink, or extend the tissue, such as fetal grasping and external pressures [167]. The UC consists of three blood vessels (a vein and two arteries), a thick supporting extracellular matrix (Wharton's Jelly), and a simple amniotic epithelium that encases the tissue. The UC vein takes nutrient-rich blood from the placenta to the fetus, and the two arteries return nutrient-depleted blood to the placenta (Figure 1C) [168, 169]. The arteries do not have an elastic membrane or an adventitia to protect the arterial integrity and blood flow, rather they are protected by smooth muscle cells and a mucoid connective tissue called the Wharton's Jelly [168–172]. This thick connective tissue constitutes the majority of the umbilical cord and has become an interesting point of discussion in regenerative medicine, and as such is the focus of this section. At term, the Wharton's Jelly is divided into three zones: the perivascular Wharton's Jelly, the intermediate Wharton's Jelly, and the subamnion [167]. These zones are characterized by the differentiation stages of the cellular populations and structure of the ECM [167]. The high concentration of ECM components in Wharton's Jelly, like water-soluble proteoglycans and HA, mixed with collagen structures, interact with water resulting in unique biomechanical properties that protect the blood vessels from compression [173]. Surrounding the entirety of the UC is an epithelial layer lining, or the “amnion” of the UC, which is contiguous with the epithelial layer of the PM and contributes to the stability of the UC by protecting it from mechanical forces in the womb [174].

The development of the UC occurs alongside that of the amnion. The precursor UC, called the body stalk, connects the embryo to the developing placenta. Around day 18 of gestation, the amniotic cavity expands and surrounds and covers the body stalk. During the first trimester, the UC lengthens, pushing the embryo further into the amniotic sac [168]. Blood flow has been documented in the UC around the end of the 5th week of gestation [175, 176]. At term, the UC length can vary between 35 – 70 cm long and 1 – 2 cm in diameter [167, 174, 177].

### 4.2. Physiology

The UC connective tissue is structured to protect the integrity of the blood vessels so as to maintain constant blood flow to the fetus [174]. The smooth muscle cells located in the arterial media facilitate contraction and blood flow in place of elastin, and the Wharton's Jelly provides a rigid structure to encase the arteries and protect vessel integrity [178]. The Wharton's Jelly also physically assists in the UC arterial contractions, regulating the pulsations that drive blood flow to the fetus [179, 180]. The contractibility of the smooth muscle cells in the vessel walls is provided by endocrine mediators like serotonin, angiotensin, and oxytocin, as well as paracrine mediators such as prostaglandins [100].

The structure of the Wharton's Jelly is honeycomb in nature, composed of a network of interconnected collagen pores that contain water-binding GAGs [169]. This porous structure allows for the movement of the mucoid ground substance as the UC contracts and expands during blood vessel contractions. These compartments also allow for the diffusion of signaling components from the Wharton's Jelly to other areas such as the amniotic cavity [181]. The composition of the Wharton's Jelly is not homogeneous and, therefore, the mechanical properties across the tissue vary. The porosity at the fetal end of the UC is higher than the maternal side [182]. Young's modulus, measuring elasticity, and the Aggregate modulus, measuring stiffness, of Wharton's Jelly is higher on the fetal side of the UC, indicating that this area of the tissue resists deformation, likely due to the smaller pore sizes at the fetal end [183]. The behavior of these smaller pores resisting flow is called poroelastic behavior and is aided by hydrophilic molecules, such as HA and proteoglycans [169].

The Wharton's Jelly is sparsely populated with cells and investigation of the distribution of cells in the Wharton's Jelly by electron microscopy and immunohistochemical staining shows that Wharton's Jelly contains cells at various levels of differentiation from fibroblasts towards myofibroblasts [184]. The density of cells is higher around the UC vessels and lower at the epithelial lining [172, 178]. Several cell types have been identified in the UC tissue, including myofibroblasts, stromal cells, mast cells, and MSCs, which are responsible for maintaining the Wharton's Jelly ECM [167, 179, 185, 186].

### 4.3. Structural Composition

Wharton's Jelly ECM is abundant and consists mainly of collagens (over 500mg/g tissue), proteoglycans, and GAGs, like HA and heparan sulfate, immobilized and imbedded in the collagen network, which provides structural support and maintains the mucoid ground substance (Table 1) [187, 188]. The proportional relationship of collagen type I (47%), III (40%), V (12%), and other collagens in Wharton's Jelly shows that collagen type I and III are the most abundant in this matrix and their proportional concentration is consistent with that of other soft tissues which typically report a higher amount of collagen type I than collagen type III [188]. Collagen is recruited to increase the stiffness of the tissue [173]. Proteoglycans, such as chondroitin sulfate, dermatan sulfate, heparan sulfate and keratin sulfate, also help provide structural support to Wharton's Jelly by regulating cellular interactions, and modulating the organization of the ECM matrix as it undergoes remodeling during gestation [189–191]. Wharton's Jelly does not contain elastin fibers, so under low strains the GAGs contribute to the elasticity of the material and the ability to retain shape under high strain [186, 189]. Up to 70% of the GAG content in UCs is HA and these high concentrations (approximately 4mg/ml) give the tissue a hydrophilic nature [188, 192, 193].

### 4.4. Cytokines and Antimicrobial Components

The ECM of Wharton's Jelly is embedded with a variety of growth factors (Table 2), including aFGF, bFGF, IGF-1, PDGFs, EGFs TGF-*β*1, IL-1, MMPs, and TIMPs [186, 194]. These signaling factors are vital to cell migration, proliferation, differentiation and remodeling of the UC ECM to maintain mechanical stability, elasticity and resistance to compression necessary to protect the UC vessels [186]. The low number of cells, especially myofibroblasts, present in Wharton's Jelly are especially stimulated to synthesize and regenerate the ECM of the Wharton's Jelly [195]. Reservoirs of signaling molecules are distributed throughout the ECM to be easily accessible to cells that regulate the tissue remodeling process of the ECM and vessels [172, 186]. For instance, high levels of MMP-2 drive the UC vessel reconstruction during gestation [196, 197]. Sulphated GAGs present in the Wharton's Jelly, such as heparin sulphate and heparin found in proteoglycans, bind cytokines and growth factors to the ECM [195]. Binding to proteoglycans protects these signaling molecules from degradation via proteolysis, creating a stable reservoir available for remodeling [195]. Studies have reported that growth factors, such as bFGF, TGF*β*, and IGF, are embedded close to these cells and their release from the ECM during matrix remodeling stimulates the surrounding cells [195, 197, 198].

Proteomic analysis of the Wharton's Jelly has identified cytokines of interest to researchers for regenerative medicine applications, such as anti-inflammatory cytokines and antimicrobial components [194]. Pro-inflammatory cytokines and anti-inflammatory cytokines work in a cascade to respond to tissue damage by removing foreign bodies through the inflammatory response before anti-inflammatory cytokines rapidly reduce inflammation and potential scarring [194, 199]. The anti-inflammatory cytokine IL-1ra is present in Wharton's Jelly, competing with IL-1 to prevent cell changes and prevent inflammation [194, 200]. Other immunomodulatory cytokines found in Wharton's Jelly, such as RANTES, IL-6R, and IFN-*γ*, play a vital role in the regulation of T cells, the immune response, macrophage activation and contribute to bacterial immunity in fetal tissues [194, 201]. Although further investigation is needed for a complete proteomic profile of UC tissue, initial studies suggest there is a large concentration of cytokines of interest for applications such as cartilage regeneration and tendon repair [186, 194, 195, 202].

### 4.5. Clinical Applications and Uses in Regenerative Medicine

UC tissue has found applications in regenerative medicine as a tissue graft and scaffold [203, 204]. The structure of the UC tissue provides an ECM scaffold for successful cell attachment and a reservoir of growth factors that play a role in tissue regeneration and cellular proliferation, as demonstrated by work with Wharton's Jelly and bone marrow MSCs and osteocytes [186, 205]. The composition of native Wharton's Jelly is attractive for wound healing applications because its natural ECM scaffold can overcome the shortcomings of other synthetically designed scaffolds that do not provide an ideal microenvironment [206]. UC tissue has been investigated for use in multiple applications, including wound healing, cardiovascular repair, cartilage repair, and treatment of diseased joints [3, 99, 204, 207–209]. Chemically stabilized umbilical cord vessels are popular for vascular reconstruction because of their length and they are often chosen over synthetic alternatives [3]. The configurations of UC tissue being researched are varied, including Wharton's Jelly sheet configurations of varying thicknesses, UC electrospun scaffolds, powderized configurations mixed with amnion material, and hydrogel compositions [205, 206, 209–211]. The processing methods that have been investigated are equally varied, including cryopreserved, lyophilized, cryomilled, electrospun, and combinations with pharmaceutical vehicles [206, 212, 213].

Clinical applications of cryopreserved, devascularized UC have been used for the treatment of DFU, with complete wound closure and re-epithelialization in the majority of patients and decreased development of scar tissue, linked to the downregulation of TGF-*β* [211]. In vitro, the extracted soluble components of a lyophilized UC sheet promoted human dermal fibroblast wound closure and the promotion of angiogenesis, and in an in vivo rat model the lyophilized, devascularized UC grafts were shown to resorb into the wound beds within 3 months [207]. A study evaluating the potential of UC tissue use in cartilage repair applications characterized Wharton's Jelly and drew parallels between its composition and that of native cartilage [204]. Decellularized and electrospun Wharton's Jelly scaffolds were investigated in cartilage tissue engineering, demonstrating desirable mechanical properties, biocompatibility, and support for cell attachment [206]. Wharton's Jelly has also been combined with other fetal tissues such as amnion in micronized configurations that can be resuspended for injection [209]. These matrix injections have been targeted for use in joint spaces where the disease state involves a decrease in the concentration of proteoglycans and damage to the ECM [99, 208]. In a rat model, knee joints injected with umbilical cord-amniotic membrane matrix saw a reduction of disease progress and reduced inflammation with no immune response, attributed to the presence of interleukins, such as IL-4, which inhibit the breakdown of proteoglycans, as well as IL-10 signaling the biosynthesis of new proteoglycans and collagen type II [209].

## 5. Placental Disc

### 5.1. Anatomy and Development

The placental disc is the transient organ that exists to support fetal development through the exchange of nutrients between maternal and fetal bloodstreams (Figure 1D). To maintain an immunological barrier, the placental disc is structured to keep the blood streams separate while maximizing surface area for the transport of nutrients between them. The placental disc begins to develop from the embryonic blastocyst when it implants into the maternal endometrium after fertilization [214–217]. The outer cells of the blastocyst develop into the placental trophoblasts, which invade the maternal tissue [214–216]. These fetal trophoblasts eventually form the fetal portion of the placental organ [214, 215]. At term, this is comprised of the chorionic plate with vascular protrusions, called chorionic villi, that extend into uterine tissue [214–217]. The UC vessels insert into the chorionic plate where they branch out into a network of vessels and capillaries in the villous structures [2]. Mature chorionic villi consist of vessels supported by a mesenchymal core, basement membranes, and a tissue layer encapsulating each villus made up of a syncytiotrophoblast layer, a cytotrophoblast layer, and an ECM [215, 218]. Trophoblast cells, mesenchymal cells, and endothelial cells of vessels are the main cell types of the placental disc [2, 215]. The syncytiotrophoblast cell layer results from the differentiation of cytotrophoblast cells creating a barrier between the maternal and fetal compartments [214–216, 218].

The maternal side of the placental disc consists of the basal plate, formed from decidual endometrial tissue [2]. Maternal blood flow is supplied by radial arteries of the myometrium that are supplied from ovarian and uterine vasculature [219]. This is pumped into the intervillous space between the basal and chorionic plates, washing the chorionic villi in maternal blood for the active and passive diffusion of gases, proteins, and nutrients

As pregnancy progresses, the placental disc grows to increase surface area to accommodate the growing demands of the fetus, allowing for additional blood flow. This is also marked by a reduction in the cytotrophoblast layer, resulting in a single syncytiotrophoblast layer between fetal and maternal blood flow [219]. At term, the placental disc is 15 to 20cm in diameter, 2 to 3cm thick and weighs approximately 500g. This gives term placental discs almost 15m^2^ of surface area for exchange between mother and fetus [219].

### 5.2. Physiology

The placental disc is the site of exchange for gases and metabolites between maternal and fetal blood, and the secretion of hormones for the support of healthy pregnancy. Substances in the maternal blood pass from the intervillous space, through the syncytiotrophoblast layer, the fetal connective tissue, and finally the fetal capillary endothelium to enter the fetal blood stream [219, 220]. Fetal lungs are not utilized for gas exchange until after birth, so all exchange of oxygen and carbon dioxide goes through the placental organ [219, 221]. Oxygen and carbon dioxide molecules are small enough to pass through the chorionic villi and fetal capillaries via passive diffusion, relying on their partial pressure gradients between the intervillous space and umbilical arteries [219, 221]. Key metabolites required for fetal development pass through the placental disc, including fatty acids, amino acids, water, electrolytes, and glucose [220, 222]. While water and electrolytes can mainly pass the placental barrier by passive diffusion or the aid of simple water channels in the trophoblasts, other metabolites require carriers, enzymatic cleavage before transfer, or facilitated transfer [219, 222–225]. For example, as fetal gluconeogenesis is limited, a high demand for maternal supplied glucose is needed, which requires the active use of glucose transporters [219, 224, 226].

The placental disc also serves an endocrine and immunological function, producing hormones and passing maternal immunity to the fetus. The syncytiotrophoblast layer acts as the endocrine tissue of the placental disc, which secretes human chorionic gonadotropin (HCG), human chorionic somatomammotropic hormone, human growth hormone variant, progesterone, and estrogens [219, 227–229]. These support pregnancy by stimulating metabolite production and enabling uterine and placental growth [219, 229]. To provide initial passive immunity to the fetus, the placental disc serves an important immunological role, allowing for the transport of maternal IgG class antibodies to the fetus [230]. These IgG antibodies are transported into the fetal blood stream via pinocytosis. IgG antibodies bind to the syncytiotrophoblasts, where they are then enclosed in vesicles and transported to the fetal blood stream to provide initial immunity for the fetus [231]

A major function of the placental disc is to maintain immune privilege and prevent rejection of the fetal tissue by the maternal immune system. One way this is achieved is by the low number of MHC molecules present in the tissue. No classical MHC class I or II antigens are found on the syncytiotrophoblast cells, in direct contact with maternal tissue [217]. Other cells of the placental disc express very low amounts of classical MHC molecules (HLA-A, HLA-B, and HLA-C), preventing the maternal immune system from recognizing the cells as not “self” [2]. The invasive cytotrophoblasts do have nonclassical class I HLA-G molecules, which have been hypothesized to prevent rejection from the mother by acting as the “universal “self” transplantation antigen” [217].

### 5.3. Structural Composition

The placental disc ECM is heavily structured in collagens, fibronectin, laminin, proteoglycans and GAGs (Table 1). The structure has a strong, organized collagenous skeleton to mechanically support the chorionic vessels and the trophoblast cell layer [232]. The basement membrane of the chorionic vessels and the stroma of the chorionic villi have a dense ECM that provides mechanical support and structure for the growth and differentiation of chorionic cells [232–234]. The villi structure is a continuous matrix of collagen fibrils and bundles woven together in different layers [232]. The structure of the network is dependent upon the stem size, thickness, and cell population [232]. The villi basement membranes contain the characteristic collagen type IV as well as collagens type I and III [233]. Additionally, collagens types I, II, III, IV, V, VI, and XIV have been extracted from human placental disc (Table 1) [232, 235]. Collagen XIV binds strongly to basement membrane-derived heparan sulfate and may be relevant in situ [235]. The trophoblasts are in contact with collagen throughout the life of the placental disc where it helps regulate trophoblast function [232]. The chorionic plate ECM plays a role in mediating development where collagen type IV has been shown to function in the differentiation of mesodermal cell lineages [232].

The placental disc contains high levels of insoluble fibronectin in the ECM [236]. Fibronectin promotes cell adhesion and has been shown to promote cell migration in culture and plays a role in the organization of the placental disc [236, 237]. Fibronectin has been particularly studied for its interaction with proteoglycans, for instance, binding to heparan sulfate in the placental disc [238]. This is structurally distinct fibronectin from the forms found in AF and maternal plasma [239]. Placental-derived fibronectin can bind twice as much carbohydrate as the plasma type, increasing its resistance to protease degradation and regulating cell interactions with tissue and other cells [240]. The fibronectin composition of chorionic villi changes through gestation and fibronectin is thought to play a role in the development of the placental tissue [239]. Higher fibronectin concentrations in early pregnancy correspond with the rapid proliferation rates of the cytotrophoblast cell column and shell in early villi development [239]. It's postulated that the trophoblasts produce less fibronectin as they mature, with more fibronectin being produced by the fibroblast and endothelial cells of the villi core [239].

Laminin is present in placental disc basement membranes of the villous trophoblast, fetal capillaries, and maternal portion of the placental disc [218, 234]. As villi develop, laminin becomes more concentrated at the villi mesenchymal cores where it contacts the trophoblast cell layer [235]. Additionally, an array of GAGs has been detected throughout placental disc tissue, including HA, dermatan sulfate, chondroitin 6-sulfate, small amounts of heparan sulfate, and large amounts of chondroitin 4-sulfate [241]. GAGs are bound or associated with the collagens of the basement membranes and fibronectin [241]. The large concentration of heparan sulfate is likely due to the extensive vascularity of the placental disc, as it is found in vessel walls [241]. Many roles have been ascribed to these molecules in utero, including providing electrochemical barriers to immunocompetent cells at the maternal sinuses and mesenchymal core, preventing structural compression of the placental disc due to the high water content held by these molecules, and acting as endogenous inhibitors of thrombin, preventing coagulation of blood and the loss of nutrient exchange [241, 242].

### 5.4. Cytokines and Antimicrobial Components

The majority of known cytokines have been detected in the placental disc and associated membranes (Table 2); however, the concentrations in the placental disc are known to vary with the progression of pregnancy and are not well characterized [243, 244]. The host of cytokines produced by the placental disc help modulate the immunological processes required for implantation, placental growth, and pregnancy [243]. Resident placental disc cells, including trophoblasts, endothelial cells, and stromal cells, secrete hormones and cytokines to support tissue metabolism, endocrine function, placental proliferation, angiogenesis, apoptosis, trophoblast invasion of the maternal tissue, and to help prevent rejection of the fetal tissue [244, 245]. For instance, a major role of the placental disc is to continually develop vasculature and villi to support fetal development. Placental growth factor (PlGF) is produced by trophoblast cells specifically and functions with VEGF, PDGFs, and leptin to promote vascular development [244, 246]. MMPs are also drivers of remodeling of the spiral arteries of the placental disc during gestation;, they are responsible for the migration of cytotrophoblasts, and the implantation into the maternal wall [56]. The placental disc also acts as a microbial barrier between fetal and maternal tissues, expressing an array of antimicrobial peptides that interplay with those expressed by the fetus and maternal cells to provide broad-spectrum microbial protection [247]. These include elafin/trappin-2, bactericidal/permeability-increasing protein (BPI), SLPI, HBD2, acyloxyacyl hydrolase (AOAH), and cathelicidin (CAP18) [247, 248].

### 5.5. Clinical Applications and Uses in Regenerative Medicine

Recent interest in the placental disc as a biomaterial has increased due to its unique function, physiology, biochemical composition and immune privilege [1, 2, 234]. This material translates well into the regenerative medicine space because of its low immunogenicity and structural and signaling components which are advantageous to the wound healing process [3]. Additionally, placental discs yield a large amount of material that can be manipulated into different tissue product configurations, processed to extract ECM components, and combined with other materials to yield gels and devices [234]. Researchers have investigated potential therapeutic applications of different components of the placental disc for several decades [2]. These include applications in the treatment of wounds, RA intervertebral disc degeneration, as well as in surgery, neurology, gynecology, and dermatology [2]. Processing of placental disc to obtain useable, clean extract involves decellularization and additional processing to obtain an extract of desired proteins, enzymes, fatty acids, and other metabolites useful in the wound healing process [249]. These processes have been developed to retain the majority of ECM and cytokine content of the cells [234]. Pharmaceutically prepared placental disc extracts have been shown to enhance the proliferation of fibroblasts and cord blood cells in vitro [2]. Animal studies have shown these placental disc extracts reduce concentrations of free radicals and inflammatory cytokines, as well as increase the proliferation of progenitor cells in vitro [2]. Use of a human placental disc hydrolysate in ligament healing of a rodent model showed marked anti-inflammatory effects, though did not impact structural characteristics of the ligament [250]. Similarly, a pharmaceutical hydrolyzed extract had antioxidative and anti-apoptotic effects, having a positive impact on skin flap survival in a rat model [251]. The presence of TGF*β*, VEGF, and FGF in placental disc extracts are associated with the increased rate of epithelialization, amplification of angiogenesis, and reduction in pain when treating non-healing wounds and burns [2]. The use of placental disc-derived ECM as a dermal substitute has also been investigated because of the similarities in cellular arrangement to that of skin [252]. Resulting studies showed that the homogenized, decellularized substitute modulated dermal healing and promoted the growth of new cells [252]. The ECM composition of the placental disc has been shown to provide the ground material for the adhesion and proliferation of cells [253].

Hydrogels prepared from isolated placental disc ECM have been shown to retain the high collagen, laminin, glycoprotein, and growth factor content of the native placental disc and to support cardiomyocytes in culture and blood vessel formation by endothelial cells, which is associated with the regenerative, pro-angiogenic, and mitogen characteristics of the ECM components [254]. Placental disc extracts combined with other materials, like silk fibroin, have shown success in a rat model for full-thickness wounds, demonstrating enhanced angiogenesis, granulation tissue formation, and re-epithelialization, resulting in improved epidermal-dermal junctions compared to a collagen-incorporated silk fibroin [255]. There has also been interest in using the placental disc ECM as a scaffold to generate organs and tissues in vitro and in vivo due to the matrix and vasculature structure of the placental disc [256, 257]. Initial results show that the placental disc framework successfully supported transplanted liver fragments and the structure is able to re-endothelialize with adjacent cells [256, 257].

## 6. Conclusion

Placental tissues are a rich, varied, and sustainable source of healthy ECM and soluble biochemical components. These tissues are available from a healthy, screened population and are otherwise treated as medical waste. Research and clinical usage of these tissues since the turn of the 20^th^ century demonstrate the current and future potential of these materials within regenerative medicine applications.

Evaluation of the composition of these native placental tissues reveals many similarities in the variety of signaling components that regulate inflammation, angiogenesis, tissue remodeling, and immune function. The varied structural compositions of these tissues have led to a wide array of configurations, suited to many regenerative applications. This in-depth comparison of placental tissue types can offer a roadmap to the selection of a placental biomaterial most suited to new applications. The placental membrane is a thin sheet with varying layers of collagens, laminins, elastins, proteoglycans, and GAGs. Amniotic fluid is another distinctive choice as a biomaterial that has the advantage of being an immune privileged fluid with soluble regulatory components and cellular constituents. The available material from the UC structure provides a scaffold rich in HA and collagens with unique biomechanical properties that can be advantageous in tissue engineering. The placental disc provides a high volume of collagens, fibronectin, laminin, and a high concentration of heparin sulfate that can be configured in varied formats.

While the use of placental membrane in wound applications is well established, novel configurations of placental tissue for a wide array of clinical applications are increasingly being investigated and implemented in recent years. Consistently, results from placental tissue use in animal models and clinical studies have shown therapeutic impacts, attributed to the combination of collagens, fibronectins, proteoglycans, GAGs, cytokines, and growth factors contained within these tissues. Increasingly, these tissues represent a unique raw material source for the development of innovative configurations for use in regenerative medicine.

## Figures and Tables

**Table 1:** Extracellular matrix components of placental membrane, amniotic fluid, umbilical cord Wharton's Jelly and placental disc.

Extracellular Matrix Components of Placental Tissues				
ECM Component	Placental Membrane	Amniotic Fluid	Umbilical Cord	Placental Disc
Collagen I	[26, 28]	[109, 127]∗∗	[188]	[232, 233, 235, 254]
Collagen II	[28]	NR	[205]	[235]
Collagen III	[28]	[109, 127]∗∗	[188]	[232, 233, 254]
Collagen IV	[28]	[108, 109]∗∗	[188]	[3, 232, 254]
Collagen V	[28]	NR	[188]	[232, 235]
Collagen VI	[28]	NR	[205]	[235]
Collagen XII	[28]	NR	[205]	[254]
Collagen XIII	[28]	NR	NR	[254]
Collagen XIV	[28]	NR	[258]	[235, 254]
Collagen XX	NR	NR	NR	[254]
Collagen XXVIII	NR	NR	NR	[254]
Aggrecan	[28]	NR	NR	[254]
Agrin	[259]	NR	NR	[254]
Chondroitin sulfate proteoglycans	[260]	[135]∗∗	[191]	[254]
Elastin	[28]	NR	NR	[254]
Fibrinogen	NR	NR	NR	[254]
Fibrillin	[28]	NR	[261]	[3]
Fibronectin	[28]	[127]∗∗	[262]	[3, 232, 235, 236, 238, 240, 254]
Ficolin-2	NR	NR	NR	[254]
Heparin sulfate proteoglycan	[12]	[135]∗∗	[188]	[3, 254]
Hyaluronic acid	[12]	[135]∗∗	[194]	[241]
Dermatan sulfate	[260]	[135]∗∗	[263]	[241]
Chondroitin 6-sulfate	[264]	[135]∗∗	[188]	[241]
Chondroitin 4-sulfate	[264]	[135]∗∗	[188]	[241]
Laminins alpha 1, 2, 3, 4; beta 1, 2; gamma 1, 3	[43] Laminin a2, a3, a5 [43] a1, a4: ND[43] b1-3, gamma 1-2 [43] gamma 3: ND	NR	NR	[3, 218, 232, 235, 254]
Nidogen	[265]	NR	ND	[254]
Vitronectin	[28]	NR	NR	[254]
Osteopontin	NR	NR	NR	[254]

∗∗ Levels are from second trimester AF collection and measurements; NR = not reported; ND = not detected.

**Table 2:** Signaling components of placental membrane, amniotic fluid, umbilical cord Wharton's jelly and placenta.

Signaling Components of Placental Tissues					
Functional role in regenerative medicine	Cytokine	Placental membrane	Amniotic fluid	Umbilical cord	Placental disc
Angiogenic signal	TGF -*β*2	[266]	[267]∗∗; [268]∗∗(TGF- *β*1 and - *β*2)	[269]	[3, 217, 243, 245, 252, 254, 270]
	TGF-*α*	[266]	[271]	[272]	[273]
	Angiogenin	[51]	[274]∗∗	NR	[252]
	VEGF	[26] VEGF-A; [26, 275–277] ND (VEGF-D)	[142]∗∗; [278]	[194](VEGF-A); NR (VEGF-B); NR (VEGF-C); NR (VEGF-D)	[3, 244, 245, 252, 254, 270]
	PlGF	[51, 58]	[279]∗∗	NR	[245, 246, 252]
	FGF-1 (aFGF)	[51]	NR	[198]	[252, 254]
	FGF-6	NR	NR	NR	[252, 254]
	FGF-2 (bFGF)	[266]	[271]	[198]	[252, 254]
	HGF	[51]	[271]	[194]	[252, 254]
	PDGF-AA, -BB	[26] PDGF-AA; [51] PDGF-BB	[280]∗∗ (PDGF-BB)	[194] PDGF-AA; NR (PDGF-BB)	[244, 252, 254]
	ANG	[51]	[281]	NR	[3]
Anti inflammatory signal	IL-4	[26]	[142]∗∗; [280]∗∗	NR	[243, 245, 282, 283]
	IL-10	[26]	[142]∗∗; [267]∗∗	NR	[243, 245, 282, 283]
	IL-13	NR	[142]∗∗	NR	[243]
	IL-1ra	[51]	[142]∗∗	[194]	[243]
	IL-6	[58]	[142]∗∗; [267]∗∗;[284]	[194]	[217, 243, 244, 252, 283]
	IL-2	[51]	[285] [142]∗∗; [280]∗∗; [284]	NR	[243, 283]
	Adiponectin	[286]	[287]	NR	[244, 288]
Protease inhibitor	TIMP-1	[26]	[289], [290]	[194]	[252]
	TIMP-2	[26]	[289–291]	[194]	[252]
	TIMP-4	[26]	[290]	[292]	[293]
Antimicrobial	IFN *γ*	NR	[142]∗∗; [280]∗∗	[194]	[243, 245, 283]
	Lactoferrin	[20]	[144]	NR	[294]
	HBD-1	[20, 60]	[153]	NR	[60]
	HBD-2	[295]	[145]	[296]	[60, 248]
	HBD-3	[295]	NR	NR	[60]
	HNP-3	[297]	[298]	NR	[297]
	Elafin/Trappin-2	[297]	[297]	NR	[60, 248]
	SLPI	[297]	[200]	NR	ND [60]
Tissue remodeling	TGF-*β*1, 2, 3	[26]	[268]∗∗ (TGF- *β* 1 and *β* 2)	[186]	[3, 17, 243, 245, 252, 254, 270]
	TGF-*α*	[51]	[271]	[194]	[273]
	Activin	[38]	[299]	NR	[245]
	VEGF	[26] VEGF-A; [26, 275–277] ND (VEGF-D)	[142]∗∗, [278]	[194](VEGF-A); NR (VEGF-B); NR (VEGF-C); NR (VEGF-D)	[3, 244, 245, 252, 254, 270]
	PlGF	[33, 51]	[279]∗∗	NR	[245, 246, 252]
	FGF-1, -2, -3, -4, -5, -6, -7	FGF-1: [51]; FGF-2: [266]; FGF-3: NR; FGF-4: [33]; FGF-5: [20]; FGF-6: [20]; FGF-7: [33]	NR	FGF-1: [198]; FGF-2: [198]; FGF-3: NR; FGF-4: [300]; FGF-5: NR; FGF-6: NR; FGF-7: [194]	[252, 254]
	EGF	[33]	[271, 301]	[186]	[252, 254, 270]
	HGF	[51]	[271]	[194]	[3, 252, 254]
	IGF-1	[51]	[271, 302]	NR	[3, 252, 254]
	PDGF-AA, -BB	[26, 51]	[280]∗∗ (PDGF-BB)	[194] PDGF-AA; NR (PDGF-BB)	[244, 252, 254]
	Adiponectin	[286]	[279]∗∗	NR	[244, 288]
	IGFBP-1, -2, -3, -4	[303] ND (IGFBP-1), ND (IGFBP-2), IGFBP-3, ND (IGFBP-4)	[304], [305]	NR	[252]
	MMP-2	[55]	[55], [306]	[197]	[56]
	MMP-9	[31], [55]	[55], [125], [306]	[197]	[56]

∗∗ Levels are from second trimester AF collection and measurements; NR = not reported; ND = not detected.

## Data Availability

This article is a review article. All data can be found in the cited references.
